# Agglomerate Growth of Xanthan Gum Powder during Fluidized-Bed Agglomeration Process

**DOI:** 10.3390/polym14194018

**Published:** 2022-09-26

**Authors:** Donghyeon Lee, Byoungseung Yoo

**Affiliations:** Department of Food Science and Biotechnology, Dongguk University-Seoul, Goyang 410-820, Korea

**Keywords:** fluidized-bed agglomeration, xanthan gum, particle size distribution, rheological properties

## Abstract

Xanthan gum (XG) powder was agglomerated via a fluidized-bed agglomeration process using water and maltodextrin (MD) binder solution, after which the products were examined. The agglomerated XG samples were collected every 10 min during agglomeration (50 min) to characterize particle growth behavior. Here, we investigated the particle size distribution, morphological characteristics, and rheological properties of agglomerates obtained at different agglomeration times. The particle size gradually increased with agglomeration time from 0 to 50 min. The porous agglomerates showed rapid growth after 40 min. The particle size of the final products tended to decrease in the dry phase for 10 min due to particle attribution during drying. Using MD as a binder solution instead of water resulted in larger XG particles. The dynamic moduli (G′ and G″) of the final product with water binder were higher than those of the native powder, whereas those of the final product with MD binder solution were lower. The G′ values of the agglomerates with MD increased gradually with agglomeration time. Native XG powders exhibited small and dense particles with a smooth surface, whereas the XG agglomerates had large and porous particles with rough surfaces and became more irregular and rougher as the agglomeration progressed.

## 1. Introduction

Food gums are widely used to modify texture and stabilize emulsions and suspensions in the food industry. Among the gums, xanthan gum (XG) has been commonly used as a food thickener and stabilizer in the food system. XG powder has been agglomerated by an agglomeration process in which the raw materials exhibit a fine particle form to produce larger dry agglomerates with desirable properties [1]. In general, this process contributes to dust reduction, improves product characteristics (flowability, dispersibility, and solubility) and physical properties, and increases the homogeneity of the final products [2]. However, there have been no studies on the mechanism of particle growth of XG powder during the agglomeration process.

The fluidized-bed agglomeration (FA) technique, which is characterized by heat and mass transfer and efficient mixing on a fluidized bed, is widely used to prepare agglomerated food powders in the food industry. The commonly used top-spray FA method consists of blowing the powders upward with hot air and spraying a binder solution from the top into a fluidized bed of powders [3]. In the FA process, fine powder particles and a binder solution are thoroughly mixed to form wet agglomerates due to viscous bridges that are consolidated by the fluidization of hot air. As the binder solution evaporates, powder particles adhere together and as the interparticle bridges strengthen, the original powder undergoes a process of agglomerate growth, after which larger agglomerates are formed upon the solidification of the binder in the dry phase [2,3]. Several studies have demonstrated that the particle size and physical properties of agglomerates can vary depending on the powder characteristics, binder type, and processing condition [4,5,6,7]. 

Research on the FA of food powders as an instant product has mostly focused on dairy-based milk powders [7,8,9], fruit and vegetable powders [10,11], and cereal powders [5,12]. Recently, significant research has been conducted on the particle agglomeration of food gums which are widely used in the food industry to improve and control the rheological properties of food products as thickening or gelling agents [3,13]. Previous studies have demonstrated that agglomerated gum powders can improve the physical and rheological properties of food formulations through the FA process. Moreover, the agglomeration of gum powders is also strongly affected by the gum type, binder type, and binder concentration. Among the various studies on gum agglomerates published to date, the studies on XG agglomerates have mainly focused on their physical and rheological properties because agglomerated XG-based food thickeners are widely used for the preparation of thickened beverages for the management of dysphagia (i.e., a swallowing disorder that commonly affects the elderly, as well as patients with gastroesophageal reflux and certain types of cancer) [1,3]. Lee and Yoo [1] studied the influence of sugar binders on the agglomeration of XG used as a food thickener. The authors found that the characteristics of XG powder were greatly affected by the binder type because the internal (e.g., porosity) and external (e.g., surface roughness) characteristics of the particles continuously change throughout the agglomeration process. The particles undergo morphological changes when a binder is sprayed on the gum powder, whereby gum powders with enhanced adhesion collide and bond with each other, thus modifying the gum matrix. Through the sequential process of particle growth, the gum powders form agglomerates with various particle shapes. Therefore, research has been conducted to assess the effects of using various binders and gums during the FA process. However, very few studies have analyzed the size and morphological properties of gum particles during particle growth in the FA process. Characterizing the changes in particle size and structural properties of gum powders in a fluidized bed during particle growth would provide key insights into the mechanisms of gum powder agglomeration. Additionally, the particle sizes and morphological characteristics of the agglomerate are greatly affected by the particle growth process, and therefore understanding the effects of different parameters on the characteristics of particle growth is vital.

Although the physical and structural properties of XG powders have been studied by a few researchers [1,3], no study has been conducted to investigate the effect of the agglomeration time on the particle growth of XG powders during the FA process. Particularly, the changes in the rheological properties of XG agglomerates with agglomeration time have never been studied. Therefore, our study sought to characterize the effect of agglomeration time on the particle size distribution (PSD), morphological characteristics, and rheological properties of XG agglomerates prepared with different binder solutions.

## 2. Materials and Methods

### 2.1. Materials

Commercial fine XG powder (Jungbunzlauer Austria AG, Wien, Austria) was used to prepare agglomerate XG samples. Distilled water and maltodextrin (MD; dextrose equivalent = 14) (Daesang Co., Gunsan, Korea) were used as binder solutions during the FA process.

### 2.2. Fluidized-Bed Agglomeration

The agglomeration of XG powder was evaluated using a top-spray fluidized-bed granulator (Fluid Bed Lab System, Dae Ho Technology Co., Ltd., Hwaseong, Korea) by spraying the powders with a binder solution, as reported by Lee and Yoo [1]. XG powder (1.5 kg) was placed in the product container and fluidized using an upward-flowing air stream. The inlet air and product temperatures were 75 ± 1.0 °C and 53 ± 1.0 °C, respectively. In this study, the fluidized-bed granulator was sprayed with 1000 mL of either deionized water or 10% (*w*/*w*) maltodextrin (MD) as binder solutions. The blower and damper were set for 70% and 30%, respectively. Then, the liquid binders were top-sprayed onto the bed at a flow rate of 20 mL/min with an air pressure of 1.5 bar for 50 min. Agglomerate samples (approximately 30 g) with different agglomeration (or spraying) times were taken every 10 min using a perforated tube in the chamber wall. Each agglomerated sample without a dry phase was immediately put into an airtight jar before analysis. After the binder solution had been completely sprayed for 50 min, the sample was dried in the fluidized bed at room temperature for 10 min to obtain the final agglomerated product. Therefore, the spray and dry phases during the agglomeration process were set for 50 min and 10 min, respectively.

### 2.3. Particle Size Distribution (PSD)

The PSD measurements were obtained with a laser diffraction particle size analyzer (Mastersizer 3000E, Malvern Instruments Ltd., Worcestershire, UK). The D_10_, D_50_, and D_90_ are the particle diameters at 10%, 50%, and 90% in the cumulative size distribution, respectively. The dispersion of PSD was measured as a span index defined as (D_90_−D_10_)/D_50_. The span index is a common calculation to quantify distribution width, which was used to determine the polydispersity of the particle distribution.

### 2.4. Particle Morphology (SEM)

Particle morphology was evaluated via an SEM. Native and agglomerated powders were attached to aluminum stubs and examined using a Hitachi S-3000 N SEM (Hitachi Ltd., Tokyo, Japan), as described previously [1]. The SEM was operated at 20 kV at a magnification of 150×.

### 2.5. Sample Preparation for Rheological Measurements

The XG solutions (1%, *w*/*w*) for rheological measurements were prepared by mixing the XG powders with deionized water under constant stirring at room temperature for 1 h, as described previously [3].

### 2.6. Rheology 

The dynamic rheological measurements of XG solutions were performed at 25 °C using a Haake RheoStress 1 rheometer (Haake GmbH, Karlsruhe, Germany) in oscillatory shear using a 35 mm plate-plate geometry and 500 μm gap, as described in a previous study [3]. Storage modulus (G′), loss modulus (G″), and loss tangent (tan δ; G″/G′) were recorded in the frequency range of 0.63–62.8 rad s^−1^ at 2% strain. Viscoelastic behavior was assessed based on tan δ, with tan δ < 1 being indicative of predominantly elastic behavior and tan δ > 1 indicating predominantly viscous behavior.

### 2.7. Statistical Analysis

All the experiments were conducted in triplicate, with data reported as mean ± standard deviation. An analysis of variance was performed on the PDS data and dynamic rheological data using ANOVA and Duncan’s multiple range test of SAS (version 9.4; SAS Institute, Inc., Cary, NC, USA) at a 5% significance level (*p* < 0.05).

## 3. Results and Discussion

### 3.1. PSD and Particle Diameter

**Figure 1:**
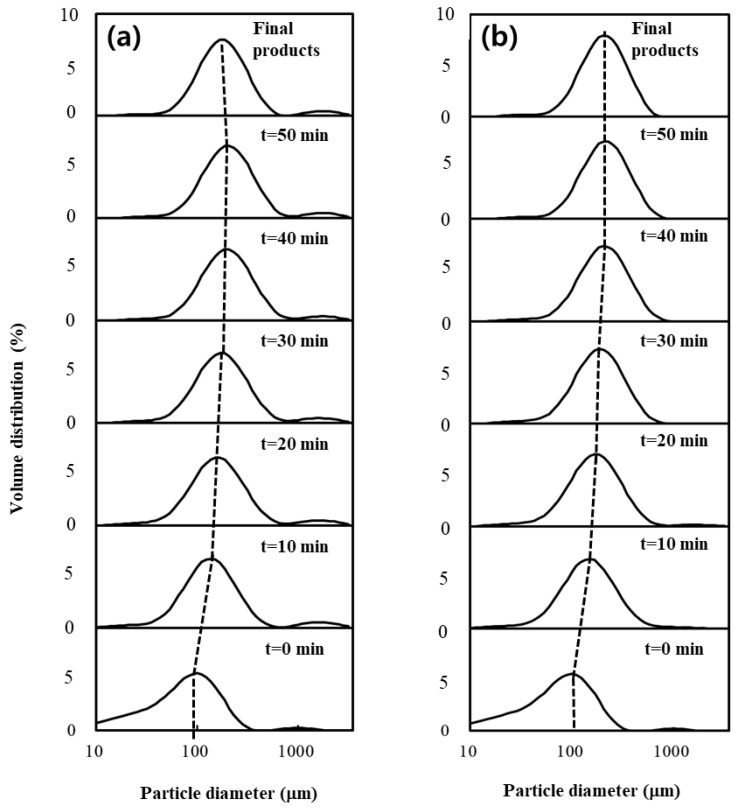
Evolution of PSD of samples taken at different agglomeration times during agglomeration with (**a**) water and (**b**) MD binders.

The mean particle diameter (D_10_, D_50_, and D_90_) and span values of the native and agglomerate XG powders with water and MD binder solution are summarized in Table 1. Figure 1 also shows the evolution of the PSD of the samples taken at different agglomeration times. The particle sizes of all XG agglomerates were significantly larger than those of the native powder, and they also increased with agglomeration (spraying) time from 0 to 50 min. This was caused by the spraying of binder solution on the XG particle surface during the agglomeration process. Particularly, the growth of XG agglomerates may be attributed mainly to a strong adhesive interaction between gum particles due to the addition of binder solution. The agglomerates at the end of the spray phase (50 min) exhibited the largest particle size compared to other agglomerates. For example, the agglomerates sprayed with water and MD exhibited a 2.1-fold and 2.0-fold increase in D_50_, respectively, compared to the native powder. However, the particle sizes (water = 189 μm; MD = 209 μm) of the final products were much lower than those (water = 227 μm; MD = 221 μm) of the agglomerates at the end of the 50 min spray phase (water = 227 μm; MD = 221 μm). In other words, the particle size was modified in the 10 min dry phase after the spraying of the binder solution had stopped. Particularly, compared to the agglomerates generated with MD, the agglomerates at 50 min generated with water showed a noticeable difference in D_50_ values relative to the final product (Figure 1), indicating that they were more friable from the shearing forces among the particles and were, therefore, more easily broken during the dry phase [2,4]. 

In contrast, the agglomerates prepared with MD remained stable throughout the dry phase due to the higher viscosity of the MD binder solution, resulting in larger particle size at the end of the agglomeration process. A similar result was observed for XG agglomerates prepared with sugar binders [1]. The span values of the XG agglomerates with water and MD during the agglomeration process were 1.61–1.91 and 1.36–1.75, respectively. Particularly, compared to the agglomerates generated with water, the span values (1.36–1.75) of the agglomerates generated with MD were much lower than that (2.09) of the native powder, indicating that MD resulted in the even agglomeration of the XG powder. In other words, MD decreased the size and volume range of the XG particles, which can be attributed to a lower disruption rate of particles due to the stronger viscous properties of the MD binder solution, as described previously [3]. Moreover, the span values (water = 1.61; MD = 1.36) of the final products were much lower than those (water = 1.72–1.91; MD = 1.46–1.75) of the agglomerates obtained at different agglomeration times. This indicates that the larger particle size of agglomerates at 50 min was reduced due to the particle attrition of these agglomerates during the dry phase, resulting in a lower variation in the particle size and volume. Therefore, the particle size of the final XG product prepared with MD binder solution appeared to be adequate, as it was possible to obtain large particles with the lowest span value compared to the final product prepared with water. Based on these results, we concluded that the PSD and particle size of XG agglomerates during the agglomeration process could be greatly influenced by the type of binder solution and agglomeration time.

### 3.2. Particle Morphology

**Figure 2:**
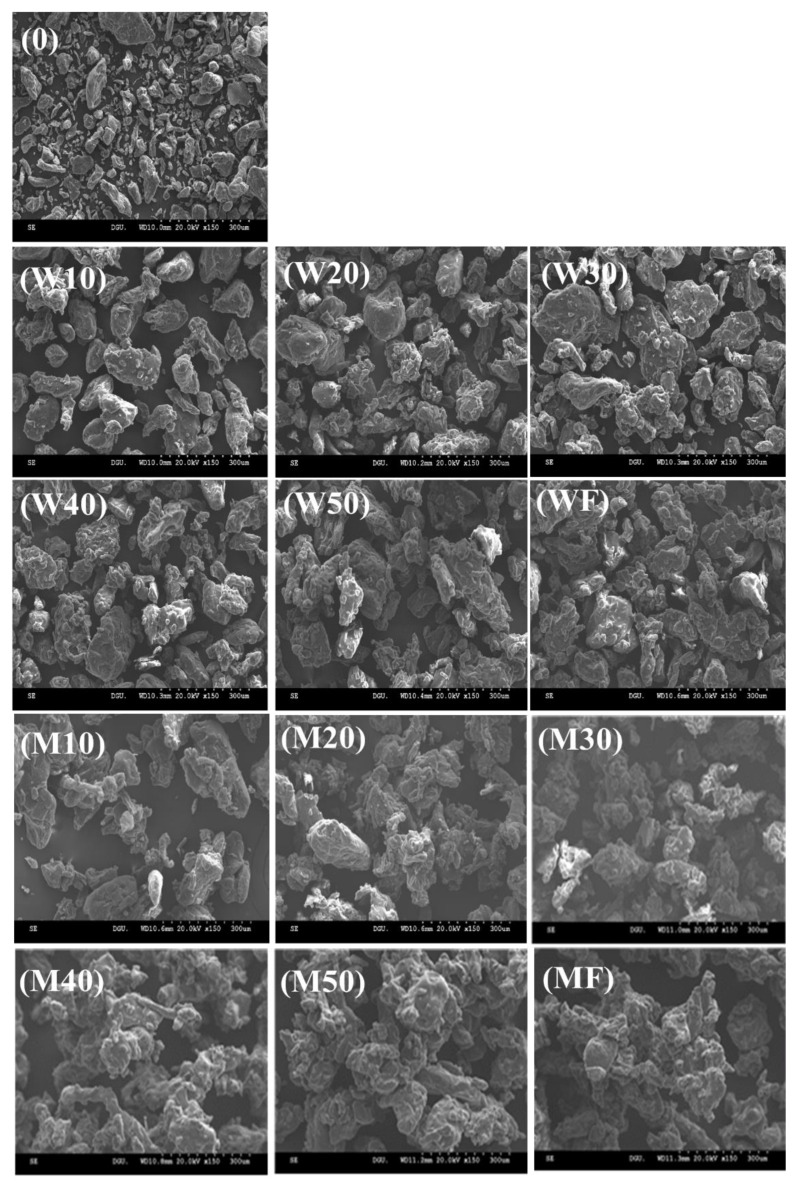
SEM images of native (**0**) and agglomerated XG powders with water (**W10**–**W50**) and MD (**M10**–**M50**) binders at different agglomeration times (10, 20, 30, 40, and 50 min): (**W10**,**M10**) 10 min, (**W20,M20**) 20 min, (**W30**,**M30**) 30 min, (**W40**,**M40**) 40 min, (**W50**,**M50**) 50 min, and (**WF**,**MF**) final powder. Magnification 150×.

Figure 2 shows the SEM images of a native powder (0), final products (WF and MF), and the agglomerates with water (W10–W50) and MD binder solution (M10–M50) at different agglomeration times (10–50 min). The native powder (Figure 2-0) consisted of small and dense particles with smooth surfaces, whereas the final products (Figure 2-WF and MF) were comprised of large and irregularly shaped particles with wrinkled and porous surfaces. Particularly, the agglomerates generated with MD binder showed a relatively greater size and porosity, as well as a more irregular shape with wrinkled and porous surfaces compared to the agglomerates prepared with water binder. This can be attributed to the higher compatibility between the XG powder and MD due to the higher viscosity of the MD solution, as described by Lee and Yoo [1]. The authors also reported that using a sugar binder solution with a higher viscosity appeared to enhance the adhesiveness of the liquid binder and XG, resulting in the formation of large particles with dense and porous structures due to the stronger intermolecular interactions between XG particles in the presence of the MD binder solution. Moreover, the particle size increased as agglomeration proceeded, and the particle shapes became rough or irregular due to a strong adhesive interaction between particles with the aid of the binder solution [1,3]. These findings indicated that the agglomeration time had a great influence on the shape and size of the XG agglomerates. Overall, the agglomeration time had noticeable effects on the shape and size of the XG powders, suggesting that the particle morphology of XG is strongly dependent on the agglomeration time.

### 3.3. Dynamic Rheological Properties

Investigating the rheological properties of gum solutions is an effective tool for estimating the occurrence of molecular interactions between gums [14]. Furthermore, the viscoelastic properties of gum agglomerates are known to be an important factor when evaluating the rheological properties of gum-based food thickeners used for the management of dysphagic patients [1]. The G′ and G″ as a function of the XG agglomerates with different agglomeration times (10–50 min) and binder solutions (water and MD) are shown in Table 2. From a structural point of view, it was found that all samples exhibited a weak gel-like behavior with tan δ values less than “1”, showing the higher G′ values than G″. In addition, there were not many differences in tan δ values between all agglomerate samples with water and MD binder solution, showing tan δ values in the range of 0.33–0.34. Such pronounced G′ and much lower tan δ values than “1” of all samples can be due to the formation of an elastic weak gel network [15]. The results also showed that the dynamic moduli (G′ and G″) values of agglomerates with water binder significantly increased with an increase in agglomeration time from 10 to 40 min, whereas those of agglomerates with MD were lower than that of native powder and did not show any tendency. Therefore, the addition of a water binder to XG powder effectively contributed to the viscoelastic properties of the agglomerates, resulting in the improved rheological quality of the XG-based food thickeners. In contrast, the addition of MD reduced the viscoelastic properties of agglomerates, showing no synergistic effect.

This result is a good agreement with other studies of various gum powders agglomerated with MD binder solution at different concentrations [3]. Particularly, the dynamic moduli (G′ = 22.4 Pa·s; G″ = 7.33 Pa·s) of the final product with water binder were significantly higher than those (G′ = 21.7 Pa·s; G″ = 7.10 Pa·s) of the native powder, whereas those (G′ = 20.2 Pa·s; G″ = 6.92 Pa·s) of the final product with MD binder solution were significantly lower. Based on these observations, we concluded that the addition of binder solution to the XG caused a slight increase or decrease in G′ and G″, which affected the elastic or viscous properties of the XG agglomerates. Therefore, we concluded that the viscoelastic properties of the XG agglomerates were greatly influenced by the type of binder solution. This tendency was previously explained by Lee and Yoo [1] who studied the dynamic rheological properties of XG agglomerates with different sugar binder solutions. 

## 4. Conclusions

To the best of our knowledge, our study is the first to investigate the effects of agglomeration times during the FA process on the agglomeration growth of XG powder, which is mainly used as a gum-based food thickener for the management of dysphagia. Spraying the XG powders with water and MD binder solution during the FA process changed the PSD, morphological properties, and rheological properties of the products. The native XG particles appeared small and smooth, whereas the surface of XG particles became rougher, and their shape became more irregular as the agglomeration process progressed. Although the particle size of XG powders increased with agglomeration time, it tended to decrease in the dry phase for 10 min after the spraying of binder solution had stopped. This suggests that the observed decreases in particle size can be due to particle attrition during the drying process in the dry phase. The addition of water binder to XG powder effectively enhanced the viscoelastic properties of the final agglomerated product, showing a synergistic effect. These results suggest that the PSD, morphological characteristics and rheological properties of XG powders can be greatly influenced by their agglomerate growths during the FA of particles, as well as the type of binder solution. Additionally, characterizing the agglomerate samples obtained during the agglomeration process could provide valuable insights into the particle growth mechanisms of XG powders. Future work will include the analysis of the agglomeration growth of XG powders in the presence of various types of binder solutions such as gums and sugars.

## Figures and Tables

**Table 1:** PSD of XG agglomerates with different binders (water and MD) as a function of agglomeration time.

Binder Type	Agglomeration Time (min)	D_10_ (μm)	D_50_ (μm)	D_90_ (μm)	Span (−)
Water	0 (Native)	15.5 ± 0.04 ^g^	72.7 ± 0.09 ^g^	167 ± 0.13 ^f^	2.09 ± 0.00 ^a^
	10	62.7 ± 1.37 ^f^ (304)	142 ± 0.92 ^f^ (95.4)	335 ± 4.12 ^e^ (100)	1.91 ± 0.04 ^c^
	20	74.8 ± 1.40 ^e^ (382)	169 ± 3.66 ^e^ (133)	411 ± 3.94 ^c^ (129)	1.99 ± 0.03 ^b^
	30	82.4 ± 0.61 ^d^ (431)	181 ± 1.88 ^d^ (149)	416 ± 8.96 ^c^ (149)	1.84 ± 0.03 ^d^
	40	96.7 ± 0.60 ^b^ (523)	209 ± 2.47 ^b^ (188)	471 ± 1.74 ^b^ (188)	1.79 ± 0.02 ^d^
	50	105 ± 1.20 ^a^ (578)	227 ± 3.83 ^a^ (212)	496 ± 0.72 ^a^ (212)	1.72 ± 0.04 ^e^
	Final product	92.0 ± 0.93 ^c^ (493)	189 ± 1.91 ^c^ (160)	396 ± 2.30 ^d^ (160)	1.61 ± 0.01 ^f^
	0 (Native)	15.5 ± 0.04 ^g^	72.7 ± 0.09 ^f^	167 ± 0.13 ^f^	2.09 ± 0.00 ^a^
MD	10	67.4 ± 0.57 ^f^ (334)	153 ± 1.63 ^e^ (110)	334 ± 3.21 ^e^ (99.5)	1.75 ± 0.01 ^b^
	20	79.9 ± 0.45 ^e^ (415)	176 ± 1.01 ^d^ (142)	357 ± 2.74 ^d^ (113)	1.58 ± 0.03 ^c^
	30	86.9 ± 0.11 ^d^ (460)	186 ± 1.92 ^c^ (156)	368 ± 4.38 ^d^ (120)	1.51 ± 0.01 ^d^
	40	99.9 ± 1.27 ^c^ (544)	211 ± 5.10 ^b^ (189)	412 ± 14.3 ^b^ (146)	1.48 ± 0.03 ^c^
	50	107 ± 0.86 ^a^ (591)	221 ± 2.55 ^a^ (204)	430 ± 5.35 ^a^ (157)	1.46 ± 0.00 ^c^
	Final product	106 ± 0.78 ^b^ (580)	209 ± 1.65 ^b^ (188)	390 ± 3.50 ^c^ (133)	1.36 ± 0.01 ^f^

The values represent the means of triplicate measurements ± SD. Means with different lowercase letters ^a–g^ within each column at each binder type are significantly different (*p* < 0.05). The numbers in parentheses represent the percentage increase in size between non-agglomerated and agglomerated gum powders.

**Table 2:** Values of dynamic rheological parameters (G′, G″, and tan δ at 6.28 rad s^−1^) of XG agglomer- ates with different binders (water and MD) as a function of agglomeration time.

Binder Type	Agglomeration Time (min)	G′ (Pa)	G″ (Pa)	Tan δ
Water	0 (Native)	21.7 ± 0.02 ^c^	7.10 ± 0.01 ^c^	0.33 ± 0.00 ^b^
	10	21.3 ± 0.01 ^e^	7.22 ± 0.02 ^b^	0.34 ± 0.00 ^a^
	20	21.5 ± 0.03 ^d^	7.24 ± 0.01 ^b^	0.34 ± 0.00 ^a^
	30	21.8 ± 0.08 ^c^	7.34 ± 0.01 ^a^	0.34 ± 0.00 ^a^
	40	21.9 ± 0.06 ^c^	7.34 ± 0.01 ^a^	0.34 ± 0.00 ^a^
	50	22.3 ± 0.05 ^b^	7.35 ± 0.03 ^a^	0.33 ± 0.00 ^b^
	Final product	22.4 ± 0.01 ^a^	7.33 ± 0.01 ^a^	0.33 ± 0.00 ^b^
	0 (Native)	21.7 ± 0.02 ^a^	7.10 ± 0.01 ^a^	0.33 ± 0.00 ^b^
MD	10	20.0 ± 0.04 ^e^	6.85 ± 0.01 ^c^	0.34 ± 0.00 ^a^
	20	21.0 ± 0.03 ^b^	6.92 ± 0.02 ^b^	0.33 ± 0.01 ^b^
	30	21.0 ± 0.02 ^b^	6.92 ± 0.03 ^b^	0.33 ± 0.00 ^b^
	40	20.3 ± 0.04 ^c^	6.75 ± 0.03 ^d^	0.33 ± 0.00 ^b^
	50	20.3 ± 0.01 ^c^	6.73 ± 0.04 ^d^	0.33 ± 0.00 ^b^
	Final product	20.2 ± 0.01 ^d^	6.92 ± 0.01 ^b^	0.34 ± 0.00 ^a^

The values represent the means of triplicate measurements ± SD. Means with different lowercase letters ^a–e^ within each column at each binder type are significantly different (*p* < 0.05).

## Data Availability

All the results showed in the manuscript could be requested to the corresponding author who would provide them.
